# Insulin Sensitivity Is Reflected by Characteristic Metabolic Fingerprints - A Fourier Transform Mass Spectrometric Non-Targeted Metabolomics Approach

**DOI:** 10.1371/journal.pone.0013317

**Published:** 2010-10-15

**Authors:** Marianna Lucio, Agnes Fekete, Cora Weigert, Brigitte Wägele, Xinjie Zhao, Jing Chen, Andreas Fritsche, Hans-Ulrich Häring, Erwin D. Schleicher, Guowang Xu, Philippe Schmitt-Kopplin, Rainer Lehmann

**Affiliations:** 1 Department of BioGeoChemistry and Analytics, Institute for Ecological Chemistry, Helmholtz-Zentrum Muenchen - German Research Center for Environmental Health, Neuherberg, Germany; 2 Central Laboratory, Division of Clinical Chemistry and Pathobiochemistry, University Hospital Tuebingen, Tuebingen, Germany; 3 Paul-Langerhans-Institute Tübingen, Member of the German Centre for Diabetes Research (DZD), Eberhard Karls University Tübingen, Tübingen, Germany; 4 Institute of Bioinformatics and Systems Biology, Helmholtz-Zentrum Muenchen - German Research Center for Environmental Health, Neuherberg, Germany; 5 Department of Genome Oriented Bioinformatics, Life and Food Science Center Weihenstephan, Technische Universität München, Freising-Weihenstephan, Germany; 6 CAS Key Laboratory of Separation Science for Analytical Chemistry, Dalian Institute of Chemical Physics, Chinese Academy of Sciences, Dalian, China; 7 Department of Internal Medicine 4, University Hospital Tuebingen, Tuebingen, Germany; 8 Department for Chemical-Technical Analysis Research Center Weihenstephan for Brewing and Food Quality, Technische Universität München, Freising-Weihenstephan, Germany; University of Bremen, Germany

## Abstract

**Background:**

A decline in body insulin sensitivity in apparently healthy individuals indicates a high risk to develop type 2 diabetes. Investigating the metabolic fingerprints of individuals with different whole body insulin sensitivity according to the formula of Matsuda, et al. (ISI_Matsuda_) by a non-targeted metabolomics approach we aimed a) to figure out an unsuspicious and altered metabolic pattern, b) to estimate a threshold related to these changes based on the ISI, and c) to identify the metabolic pathways responsible for the discrimination of the two patterns.

**Methodology and Principal Findings:**

By applying infusion ion cyclotron resonance Fourier transform mass spectrometry, we analyzed plasma of 46 non-diabetic subjects exhibiting high to low insulin sensitivities. The orthogonal partial least square model revealed a cluster of 28 individuals with alterations in their metabolic fingerprints associated with a decline in insulin sensitivity. This group could be separated from 18 subjects with an unsuspicious metabolite pattern. The orthogonal signal correction score scatter plot suggests a threshold of an ISI_Matsuda_ of 15 for the discrimination of these two groups. Of note, a potential subgroup represented by eight individuals (ISI_Matsuda_ value between 8.5 and 15) was identified in different models. This subgroup may indicate a metabolic transition state, since it is already located within the cluster of individuals with declined insulin sensitivity but the metabolic fingerprints still show some similarities with unaffected individuals (ISI >15). Moreover, the highest number of metabolite intensity differences between unsuspicious and altered metabolic fingerprints was detected in lipid metabolic pathways (arachidonic acid metabolism, metabolism of essential fatty acids and biosynthesis of unsaturated fatty acids), steroid hormone biosyntheses and bile acid metabolism, based on data evaluation using the metabolic annotation interface MassTRIX.

**Conclusions:**

Our results suggest that altered metabolite patterns that reflect changes in insulin sensitivity respectively the ISI_Matsuda_ are dominated by lipid-related pathways. Furthermore, a metabolic transition state reflected by heterogeneous metabolite fingerprints may precede severe alterations of metabolism. Our findings offer future prospects for novel insights in the pathogenesis of the pre-diabetic phase.

## Introduction

In the pathogenesis of type 2 diabetes mellitus initial metabolic alterations occur even decades before the manifestation of this epidemic lifestyle disease [1], [2]. This long asymptomatic period represents the so called pre-diabetic state. It is dominated by a gradually developing insulin resistance of skeletal muscle, liver and fat tissue as well as the dysregulation of insulin secretion. However, in contrast to the evident diabetic state the mechanisms related to the decline in insulin sensitivity, in particular the altered metabolic pathways and the transition process, are much less clear.

Metabolomics tools have been proven in several recent human studies to fulfill the prerequisite to study metabolic alterations of body fluids for the discrimination between states of health and disease [3]–[10]. Manifest type 2 diabetes has been investigated in several recent targeted- and non-targeted- (NT-) human studies [11]–[20]. Metabolomics approaches have also been taken to study the effects of anti-diabetic drugs on the metabolite pattern and the affected pathways in type 2 diabetic subjects [21], [22]. However, metabolite patterns from pre-diabetic humans have mostly been studied by targeted profiling strategies only [14], [19], [23]–[25]. NT-metabolomics investigations of plasma from pre-diabetic subjects by ion cyclotron resonance Fourier transform mass spectrometry (ICR-FT/MS) has not been carried out so far. In total, only a few applications of ICR-FT/MS analyzing metabolites in plasma samples in the flow injection mode can be found [26], [27]. Using ICR-FT/MS with high magnetic fields (12 Tesla) enables the highest resolution (400.000 at m/z 400) combined with the best mass accuracy (<200 ppb) for a direct conversion of the experimental mass into elementary compositions. Previous metabolomics studies showed that this technology together with multivariate statistics and bioinformatics allows to differentiate thousands of exact mass signals [28]. These could be directly annotated to probable metabolites and their isomers with information on metabolic pathways [28], [29] from existing databases [30].

In our NT-metabolomics ICR-FT/MS study we first aimed to group 46 non-diabetic subjects into metabolic healthy individuals and indviduals with reduced insulin sensitivity. To this end, individual metabolic fingerprints detected in the plasma by ICR-FT/MS were correlated with the insulin sensitivity index calculated according to Matsuda [31]. A second objective of our study was to identify the metabolic pathways that are most affected by the decline in insulin sensitivity. This goal was achieved by translating the detected metabolite ions into pathways using the metabolic annotation interface MassTRIX which assigns to each m/z value potential annotations from various metabolite data bases [30]. NT-metabolomics analysis by ICR-FT/MS combined with bioinformatics approaches revealed three clusters of different metabolic fingerprints, namely, an insulin sensitive group, individuals in a metabolic transition state, and subjects with a metabolic fingerprint associated with a clear decline in insulin sensitivity. Furthermore, alterations of distinct metabolic pathways were detected which offer novel insights into the switch of individual metabolite pattern along with the decline in insulin sensitivity.

## Results and Discussion

### Investigation of the metabolic conversion from normal to reduced insulin sensitivity reflected by individual metabolite fingerprints

In non-diabetic individuals a decline in whole body insulin sensitivity indicates a high risk to develop type 2 diabetes. One of the most widespread indices used in clinical and research settings to estimate whole body insulin sensitivity is the insulin sensitivity index proposed by Matsuda and DeFronzo [31] (ISI_Matsuda_) which is derived from oral glucose tolerance test (oGTT) data. It reflects a composite estimate of hepatic and muscle insulin sensitivity, which decline in the pre-diabetic metabolic state resulting in a lower ISI_Matsuda_ value [31], [32]. Of note, to the best of our knowledge only a threshold to identify diabetic subjects (ISI_Matsuda_ <2.5) can be found in the literature [32], [33], but no ISI_Matsuda_ threshold for the identification of insulin resistant pre-diabetic subjects has been determined so far. Aiming in a first step to differentiate subjects with decreased insulin sensitivity ( =  low ISI_Matsuda_) from those with normal ISI_Matsuda_ levels based on the individual metabolite patterns we applied a NT-metabolomics ICR-FT/MS-driven analysis of plasma samples leading to mass spectra containing up to 2958 metabolite ion masses per individual. We hypothesized that a relationship could exist between the set of variables (m/z values of ion masses) and the ISI_Matsuda_ values. In order to validate this hypothesis we built up a partial least square (PLS) model where the dependent variable Y was set to “Insulin Sensitivity Indices”. A three components model was found optimal using a cross validation approach provided by SIMCA-P 12 [34]. Subsequently, this model was internally validated using the permutation testing routine [35]. 200 randomly permuted models showed all less significance than the original model. Additionally, no sign of overfitting was detected.

**Figure 1:**
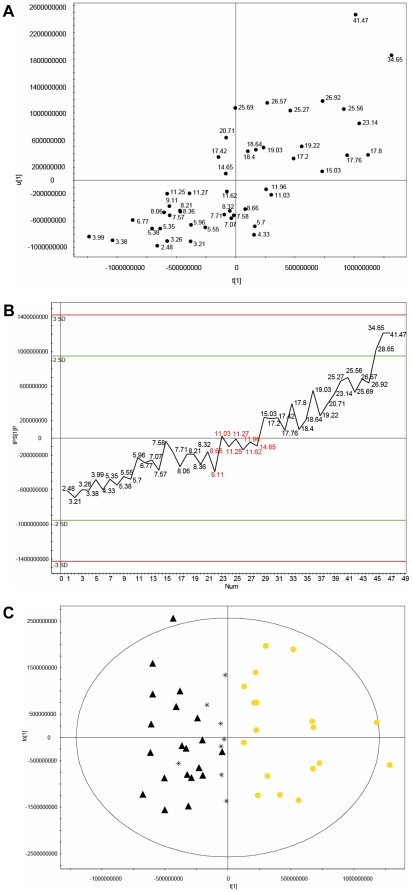
Investigation of individual metabolite fingerprints reflecting the metabolic conversion from normal to reduced insulin sensitivity. (**A**) Individual ISI_Matsuda_ values (to the right of each dot) assigned to the metabolite fingerprints in an OSC-PLS score scatter plot t[1]u[1] achieving R^2^(Y) = 0.94 and Q^2^(cum) = 0.90. (**B**) Score scatter plot of the first component tPS[1] against ISI_Matsuda_ values using the transition group as external dataset. From this visualization it was inferred that the transition group revealed properties closer to the class of subjects with reduced ISI values. (**C**) A threshold of ISI_Matsuda_  = 15 was set to generate an OPLS model showing a clustering into two groups of subjects, i.e. with normal and declined insulin sensitivity. Using CrossValidation-ANOVA the model showed a high significance (p = 4,76199×10^−19^). Furthermore, the model achieved the following parameters: R^2^(Y) = 0.96 and Q^2^(cum) = 0.93. Definition of labels: (▴) ISI_Matsuda_ level < 8.5, (*) ISI_Matsuda_ from >8.5 and <15, and (•) ISI_Matsuda_ >15.

The orthogonal signal correction-partial least-squares (OSC-PLS) score scatter plot (Figure 1A) suggests that the metabolite pattern of individuals with an ISI_Matsuda_ around 15 already differs from the one of individuals with lower ISI values. This gives a first hint that an ISI_Matsuda_ threshold for the pre-diabetic state would be considerably higher than the threshold of <2.5, which has been established for the detection of diabetic subjects [32], [33]. Next, we performed further multivariate investigations of the characteristics of the metabolic fingerprints applying an orthogonal-partial least-squares (OPLS/O2PLS) model. We detected a subgroup of eight subjects which fit in part to the metabolite pattern of the group with low insulin sensitivity, but also match to some extent to the group with normal insulin sensitivity. Therefore, we hypothesize that the metabolite pattern of these individuals (ISI_Matsuda_ value ranges between 8.5 and 15) may represent some kind of metabolic transition state from normal to reduced ISI values.

In the next step we analyzed the data for more pronounced similarities between the transition group on one hand and either the group with normal or impaired insulin sensitivity on the other. Figure 1B shows the projection of the transition samples in the next OPLS-DA model generated. The score scatter plot of the first component tPS[1] shows the prediction of the transition group, used as external dataset. From this visualization it could be concluded that the transition group shows properties closer to the class of subjects with reduced ISI values (ISI_Matsuda_ <8.5). Assuming that the transition group belongs to the class with reduced insulin sensitivity, an OPLS model was generated where only two classes were defined (ISI_Matsuda_ <15 and ISI_Matsuda_ >15). Figure 1C shows two distinct clusters with an ISI_Matsuda_ of 15 as threshold. Of note, the Cross Validation-ANOVA (CV-ANOVA) of the model had a high significance (p = 4.76199×10^−19^) and the validity of the model was also confirmed by the values R^2^(Y) = 0.96 and Q^2^(cum) = 0.93. These findings confirm the hypothesis that the metabolic patterns of the transition group are more similar to the group with decreased ISI. As a further confirmation of the ISI threshold of 15, clustering into two groups was not possible for models generated with values lower than 15, (data not shown).

### Detection of metabolic pathways reflecting conversion from normal to reduced insulin sensitivity

To screen for the pathways most affected in individuals with decreased insulin sensitivity the NT-metabolomics data were evaluated using MassTRIX [30]. The metabolite ion masses with the highest regression coefficients were considered as discriminants for the two classes. From the total of detected masses, 4847 m/z-values were selected as being typical for individuals with an ISI value <15, and 5035 for those with an ISI >15.

To annotate putative metabolites and the corresponding pathways these selected masses were uploaded into MassTRIX [30]. Only 19% of the mass signals could be assigned to metabolites; all others, including isotopologues are important unknown chemical structures (data not shown). The pathways potentially affected by differential metabolite intensities in the two groups are given in Table 1. This analysis revealed that arachidonic acid metabolism, steroid hormone biosyntheses, bile acid metabolism, metabolism of essential fatty acids (linoleic and α-linolenic acid) and biosynthesis of unsaturated fatty acids comprise the highest number of intensity differences in known detected metabolites between the two groups (Table 1). Of note, not all of these compounds are unique for the respective pathway. For example, we found 40.2% of unique compounds in arachidonic acid metabolism, 61.9% in steroid hormone biosynthesis, 40.9% in bile acid biosynthesis, 69.2% and 45%, respectively for α-linolenic acid metabolism and linoleic acid metabolism, and 38.9% in the pathway of the biosynthesis of unsaturated fatty acids.

**Table 1:** Metabolic pathways altered in subjects with reduced insulin sensitivity (ISI_Matsuda_ <15) in descending order of the number of detected differing metabolites.

Pathway description	Count diff. metabolites
Arachidonic acid metabolism (hsa00590)^a^	77
Steroid hormone biosynthesis (hsa00140)^a^	71
Bile acid biosynthesis (hsa01120)^a^	22
Linoleic and α-Linolenic acid metabolism (hsa00591, hsa00592)^a^	33
Biosynthesis of unsaturated fatty acids (hsa01040)^a^	18
Porphyrin metabolism (hsa00860)^a^	16
Retinol metabolism (hsa00830)^a^	15
Sphingolipid metabolism (hsa00600)^a^	14
Galactose metabolism (hsa00052)^a^	12
Fructose and mannose metabolism(hsa00051)^a^	11
Phenylalanine metabolism (hsa00360)^a^	10
Fatty acid biosynthesis (hsa00061)^a^	10

^**a**)^KEGG pathway number.

Pathways with less than 10 metabolites differing between individuals with reduced and normal insulin sensitivity are not shown. By comparing the exact experimental mass signals (threshold set as signal to noise ≥3) with the theoretical masses in MassTRIX, the counts of metabolites were obtained and could be assigned to the different metabolic pathways.

Alterations in the metabolism of essential fatty acids and polyunsaturated fatty acids are discussed to play a significant role in the pathophysiology of metabolic syndrome and diabetes mellitus [36]. A targeted metabolomic investigation of the plasma pattern of families burdened with early-onset cardiovascular disease revealed linoleic acid (LA) and arachidonic acid (AA) as biomarkers [37]. Bile acids, having for a long time been viewed solely as detergents that emulsify nutrient lipids, are now discussed as metabolic integrators of whole-body energy homeostasis that influence glucose and lipid metabolism [38]. In addition, bile acids were among the metabolites showing the most striking changes during an oGTT in a targeted metabolic profiling approach [14] and in a non-targeted metabolomics study recently performed by our group [17].

Since the annotated pathways of arachidonic, linoleic and α-linolenic acid as well as the biosynthesis of unsaturated fatty acids are interrelated and comprise a high number of metabolites with altered intensities (Table 1) we focussed in more detail on the metabolite ion masses of those pathways.

### Investigation of dominating pathways reflecting conversion from normal to reduced insulin sensitivity

**Figure 2:**
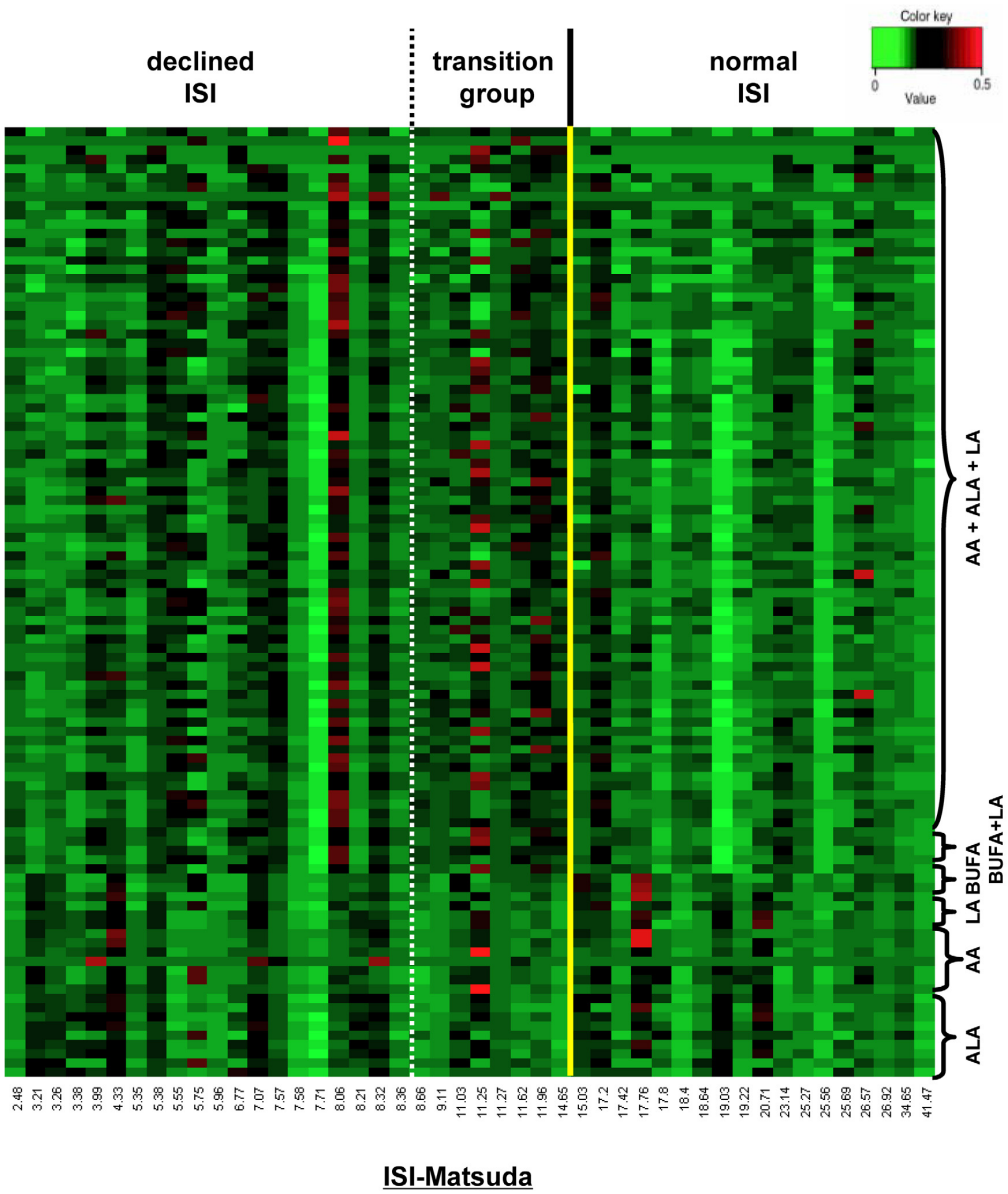
Heat map of plasma metabolites from linoleic acid (LA), α-linolenic acid (ALA), arachidonic acid (AA) pathways and biosynthesis of unsaturated fatty acid (BUFA). The heat map represents the signal intensities of 18 subjects with normal insulin sensitivity (ISI_Matsuda_ >15), eight individuals who are in the metabolic transition state and 20 individuals with declined insulin sensitivity (ISI_Matsuda_ <15). Individual ISI_Matsuda_ levels in increasing order are given in the column at the bottom. Cells are coloured based on the signal intensity measured in plasma. Shades of red to green represent high to low signal intensities of the metabolite ions in plasma (see color scale on the right side of the heat map). A yellow line is drawn at an ISI_Matsuda_ value of 15 to mark the potential ISI_Matsuda_ threshold separating individuals with altered and normal insulin sensitivity revealed in our NT-metabolomics approach. A dash dotted white line drawn at an ISI_Matsuda_ value of 8.5 marks the suggested threshold between individuals with distinct insulin resistant metabolic alterations and subjects who are in the metabolic transition state.

In a first step a metabolite heat map was generated solely based on the metabolite ion masses of the pathways of α-linolenic acid, linoleic acid, arachidonic acid, and biosynthesis of unsaturated fatty acids (Fig. 2). The heat map was divided into three sections representing individuals with declined ISI_Matsuda_, metabolic transition state and normal ISI_Matsuda_, respectively.

In normal controls green shades dominate (Fig. 2), representing low relative metabolite concentrations. In subjects with declined insulin sensitivity and in the transition group dark shaded colours predominate, representing medium to high relative metabolite concentrations. However, the heat map also reveals a high interindividual variability (Fig. 2). Particularly in the group with declined insulin sensitivity the patterns of the individuals with an ISI of 7.58, 7.71 and 8.06 were highly different from the patterns of the other individuals within this group.

**Figure 3:**
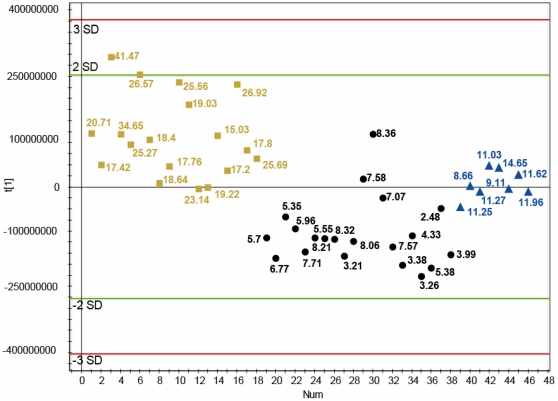
Discrimination power of the signal intensities of four selected pathways. A score scatter plot of metabolite ion masses solely related to the pathways of linoleic acid, α-linolenic acid, arachidonic acid pathways and biosynthesis of unsaturated fatty acid was generated. The classes in the score scatter plot are defined as: (•) ISI_Matsuda_ >15; (▴) ISI_Matsuda_ <8.5 and (*) transition group (ISI_Matsuda_ >8.5 and <15).

Since the heat map patterns of individuals with declined ISI_Matsuda_, metabolic transition state and normal ISI_Matsuda_ clearly showed distinct trends but also high interindividual variability we speculated about the discrimination power of these four pathways. Therefore, we further investigated the impact of these four pathways by generating a score scatter plot based solely on the metabolite signal intensities of α-linolenic acid and linoleic acid, arachidonic acid pathway, and biosynthesis of unsaturated fatty acids. Fig. 3 demonstrates a separation of the groups of individuals with an ISI_Matsuda_ <8.5 and >15, the only exception being two individuals with an ISI_Matsuda_ of 7.58 and 8.36 who show closer similarity to the group with normal insulin sensitivity. The transition group (labelled by triangles) in the score scatter plot (Fig. 3) was again identified as an intermediate state. These data reveal a potential relevance of alterations of these four pathways in the mechanistic scenario of reduced insulin sensitivity.

**Figure 4:**
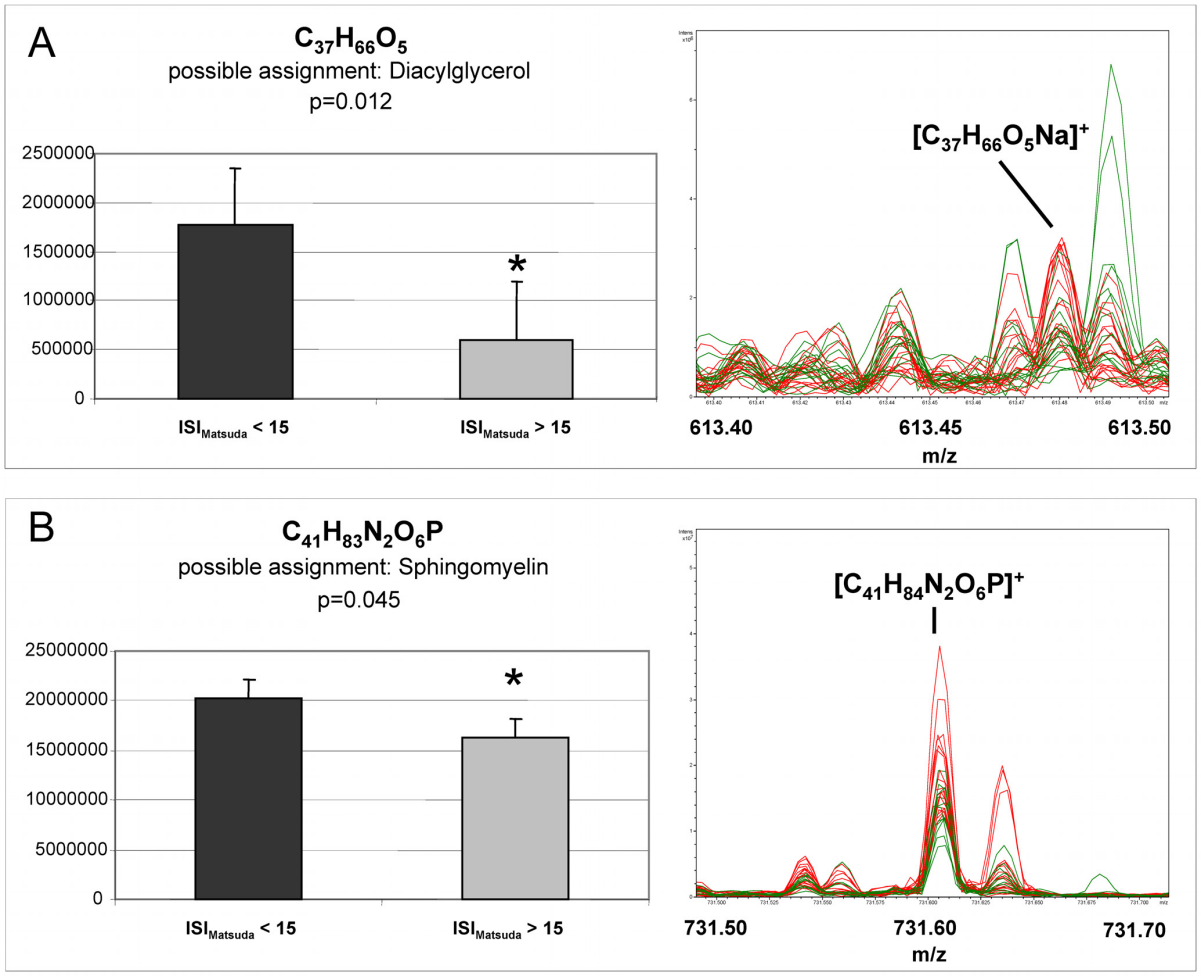
Comparison of the signal intensities of diacylglycerol and sphingomyelin in plasma of individuals with reduced and normal insulin sensitivity. A non parametric Wilcoxon rank sum test was applied on the signal intensities of the ions with the elemental composition of (**A**) C_37_H_66_O_5_ (possible assignment: Diacylglycerol) and (**B**) C_41_H_83_N_2_O_6_P (possible assignment: Sphingomyelin) to evaluate statistical significances between the two groups (ISI <15 (n =  28) *vs*. ISI_Matsuda_ >15 (n =  18)). On the right side the corresponding individual ICR-FT/MS spectra of the nominal masses are given; green  =  ISI >15 and red  =  ISI < 15. Mean peak areas ± standard error are shown, statistical significance (*) was set at p<0.05.

It is important to note that differences between means of most metabolite signals of the four pathways investigated in the heat map (Fig. 2) and the score scatter plot (Fig. 3) did not reach statistical significance applying non parametric Wilcoxon rank sum test because of great interindividual variabilities of the metabolite signal intensities. However, evaluating the total list of metabolite ion masses we detected significant differences exemplarily shown in Figure 4 for the metabolite ions with the elementary composition of C_37_H_66_O_5_ (m/z = 613.48004 for [C_37_H_66_O_5_Na]^+^; possible assignment: Diacylglycerol), and C_41_H_83_N_2_O_6_P (m/z = 731.60628, [C_41_H_84_N_2_O_6_P]^+^ possible assignment: Sphingomyelin).

The metabolite ion possibly assigned to diacylglycerol (DAG) showed significantly increased levels in individuals with reduced insulin sensitivity (ISI_Matsuda_ below 8.5) as well as in the transition group (Fig. 4A). DAG has long been discussed as a key lipid intermediate linking nutrient excess to dysregulation of insulin action [39]. A large dietary fat load may lead to accumulation of diacylglycerols and subsequently to activation of protein kinase C isoforms [40]. Furthermore, the intramyocellular concentration of DAG in skeletal muscle has been shown to increase in insulin-resistant rodents and humans [40]. Since DAG concentrations appeared to be similarly elevated in both the transition group and the insulin resistant group (data not shown), this increase could represent an early event in the development of insulin resistance.

Another example of a metabolite found to be increased in the pre-diabetic group (ISI_Matsuda_ <15) compared to the group with normal insulin sensitivity is the metabolite ion possibly assigned to sphingomyelin (Fig. 4B). Sphingomyelin is also among the candidate molecules likely involved in pathomechanisms relevant for a decline in insulin sensitivity, since the breakdown of sphingomyelin leads to the generation of ceramide which is a potent signaling molecule well known to mediate insulin resistance [39]–[41]. It appears worthwhile to verify the identity of this metabolite and to investigate its plasma concentration in larger cohorts in order to determine whether it is a transient marker for a very early state of declining insulin sensitivity.

A further interesting observation was the detection of a metabolite with the elemental composition C_33_H_42_N_4_O_6_ (possible assignment: Urobilinogen) in seven individuals with an ISI <8.5 (data not shown). This finding is of potential interest since this metabolite was neither detectable in individuals of the transition group nor in the group with normal insulin sensitivity. Urobilinogen is a product of bilirubin metabolism, formed by intestinal reduction through bacteria of the gut flora. It is mainly excreted in faeces, but a small amount is reabsorbed in the terminal ileum and colon and enters the enterohepatic circulation (called the “enterohepatic urobilinogen cycle”) from where a minor fraction is excreted by the kidney. Hence, the hepatic metabolisms as well as the gut flora and to a lesser extent the kidney are involved in its metabolism and may affect the urobilinogen plasma levels in subjects with reduced insulin sensitivity.

In conclusion, out of thousands of m/z signals obtained with the ICR-FT/MS approach we detected characteristic metabolite fingerprints in plasma of 46 individuals displaying different insulin sensitivities. These findings allowed us to divide the subjects into two groups, one with unsuspicious metabolic pattern and the other with reduced insulin sensitivity. Furthermore, the results suggest an ISI_Matsuda_ >15 as a potential threshold to discriminate between these two groups. The decline in insulin sensitivity was reflected by alterations in various metabolic pathways. However, many metabolite ion masses that are not covered by current databases still have to be elucidated to give a proper view of the whole metabolic network affected in subjects at high risk to develop future type 2 diabetes.

## Materials and Methods

### Ethics Statement, subjects and study design

The protocol (422/2002) was approved by the Institutional Review Board of the University of Tuebingen, Schleichstr. 8, 72076 Tuebingen (board chairman: Prof. Dr. D. Luft) according to the Declaration of Helsinki, and all subjects gave written informed consent before the study commenced. The investigation was conducted in accordance with the ethical principles of Good Clinical Practice. For our study group fifty individuals having fasting blood glucose levels between 4.5 and 6.5 mmol/l (mean: 5.3±0.53 mmol/l) which are all below the current diagnostic criteria of the WHO for type 2 diabetes [42], were recruited. Hemoglobin A1c (HbA1c) levels were between 4.4 and 6.5% (mean: 5.5±0.44%). Eight individuals were in the HbA1c range of 6.0 to 6.5%, recently discussed by the “International Expert Committee Report” to reflect individuals at highest risk for progression to diabetes [43]. In our study group glucose, HbA1c, age (mean: 48.3±10.3 years) and waist-to-hip-ratio (0.87±0.088) were normally distributed. After an overnight fast all individuals underwent a 75 g oral glucose tolerance test (oGTT) under standardized conditions in the metabolic ward at the University Clinic in Tuebingen (Germany) according to the recommendations of the WHO [42]. Venous blood samples were obtained at 0, 30, 60, 90 and 120 minutes of the oGTT for determination of plasma glucose and insulin. Based on the definition of the WHO concerning the 120 min blood glucose concentration during an oGTT [42] four subjects were diagnosed to have type 2 diabetes and were excluded from the study. From the remaining 46 subjects insulin sensitivity indices (ISI) were calculated applying the formula proposed by Matsuda and DeFronzo [31], henceforth referred to as ISI_Matsuda_. The ISI_Matsuda_ ranged from 2.48 to 41.47 in our study population. Glucose was measured with the ADVIA 1650 clinical chemical analyzer and insulin with the ADVIA Centaur immunoassay autoanalyzer, both analyzers were from Siemens Healthcare Systems (Fernwald, Germany). HbA1c was determined by cation-exchange HPLC (Tosoh, Germany).

### Sample preparation for the NT-metabolomic analysis

The plasma samples (n = 46) collected in the fasting state were aliquoted in volumes of 200 µl and immediately stored at −80°C. Aliquots were thawed on ice, 50 µl were diluted with 50 µl water to a final volume of 100 µl. The samples were acidified by the addition of 10 µl 25% formic acid prior to solid phase extraction (SPE). SPE was done using OMIX Tip C18 (Varian). The cartridges were conditioned by flushing the tips ten times with 100 µl methanol followed by 100 µl of water acidified with formic acid (100 µl water and 5 µl 25% formic acid). Next, the OMIX tip was flushed ten times with the acidified sample and subsequently washed one time with 100 µl water acidified with formic acid. The compounds were then eluted by flushing the tips ten times in 100 µl methanol and were ready for infusion analysis.

### Mass spectrometric procedure

High-resolution mass spectra were acquired on a Bruker (Bremen, Germany) APEX Qe Fourier transform ion cyclotron resonance MS equipped with a 12 Tesla superconducting magnet and an Apollo II source. The samples were infused and ionized by TriVersa Nanomate chip electrospray system (Advion, Ithaca, USA) in positive mode. After optimisation the chip voltage was set to 1.6 kV, and a backpressure of 0.5 psi was used. The MS sampling electrodes, consisting of the spray shield and the metal cap mounted on the high-pressure side of the transfer capillary, were both set to 0 V. A drying-gas flow of 4 L×min^−1^ at 200°C was used. The spectra were acquired with a time domain of 1 MW over a mass range of 100–2000 m/z. Two hundred fifty six scans were accumulated for each spectrum. Spectra were externally calibrated first on clusters of arginine (10 mg/l in methanol) and internally calibrated on solvents impurities (diester); calibration errors in the relevant mass range were always below 100 ppb. Data acquisition and handling were performed by using the Data Analysis Software v.3.4 (Bruker Daltonics, Bremen, Germany). Peaks exceeding a threshold signal-to noise ratio of 3 were exported to peak lists.

### Data analysis

The spectra exported from DataAnalysis were aligned through in-house software; the data were stored in a matrix by aligning all the peaks within an error of 1 ppm. The total numbers of individual variables (m/z) for all samples were 31810. In order to stabilize the variance the m/z with only one value were excluded from the calculation to reach a final dataset counting 15054 individual m/z values. The intensity for each peak was normalized to the sum of the peak intensities for each data set.

The SIMCA-P 12 (Umetrics, Umea, Sweden) software was used to visualized and define the relation between the samples through several multivariate methods. All statistical elaborations were done after an Orthogonal Signal correction (OSC), in order to remove the information that is orthogonal to Y from the independent variables (X). We combined different multivariate techniques in order to explain the relation between a reduced number of masses and the ISI_Matsuda_ value. Each model was internally validated with 7-fold cross-validation and with the permutation test.

The selection of the m/z values more related to low and high ISI values was performed based on the highest absolute value of the regression coefficients and on the variable importance in the projection (VIP). The masses responsible for the metabolic differentiation were submitted to MassTRIX using *homo sapiens* as reference species and a max. error of 3 ppm. This web server assigns to each m/z value potential annotations from KEGG (http://www.genome.jp/kegg/), LipidMaps (http://www.lipidmaps.org/) and HMDB [44]. In a second step MassTRIX then calls the KEGG/API (http://www.genome.jp/kegg/soap/) to generate pathway maps of the annotated masses.

2902 out of 15054 compounds were annotated by this approach. Out of the 100 most important m/z VIP values, 1791 compounds were recognized from MassTRIX. 81% of the m/z values remaining unknown show the need for further structural investigations with orthogonal technologies (LC/MS^2^ or LC/NMR).

The statistical analyses were done with SIMCA-P 12 (Umetrics, Umea, Sweden), SAS version 9.1 (SAS Institute Inc., Cary, NC, USA) and for the heatmap visualization with the statistical package “R” (v. 2.9.2). Statistical significance was set at p<0.05.
